# Resilience Amidst Crisis: Lessons Learned From the COVID-19 Pandemic’s Impact on Cardiovascular Fellowship Programs

**DOI:** 10.1016/j.jacadv.2024.101006

**Published:** 2024-05-24

**Authors:** Benjamin H. Freed, Doreen DeFaria Yeh, Melinda B. Davis

**Affiliations:** a Division of Cardiology, Northwestern University Feinberg School of Medicine, Chicago, Illinois, USA; b Department of Cardiology, Massachusetts General Hospital, Harvard Medical School, Boston, Massachusetts, USA; c Division of Cardiovascular Medicine, University of Michigan, Ann Arbor, Michigan, USA

**Keywords:** fellowship, pandemic, survey

**Figure:**
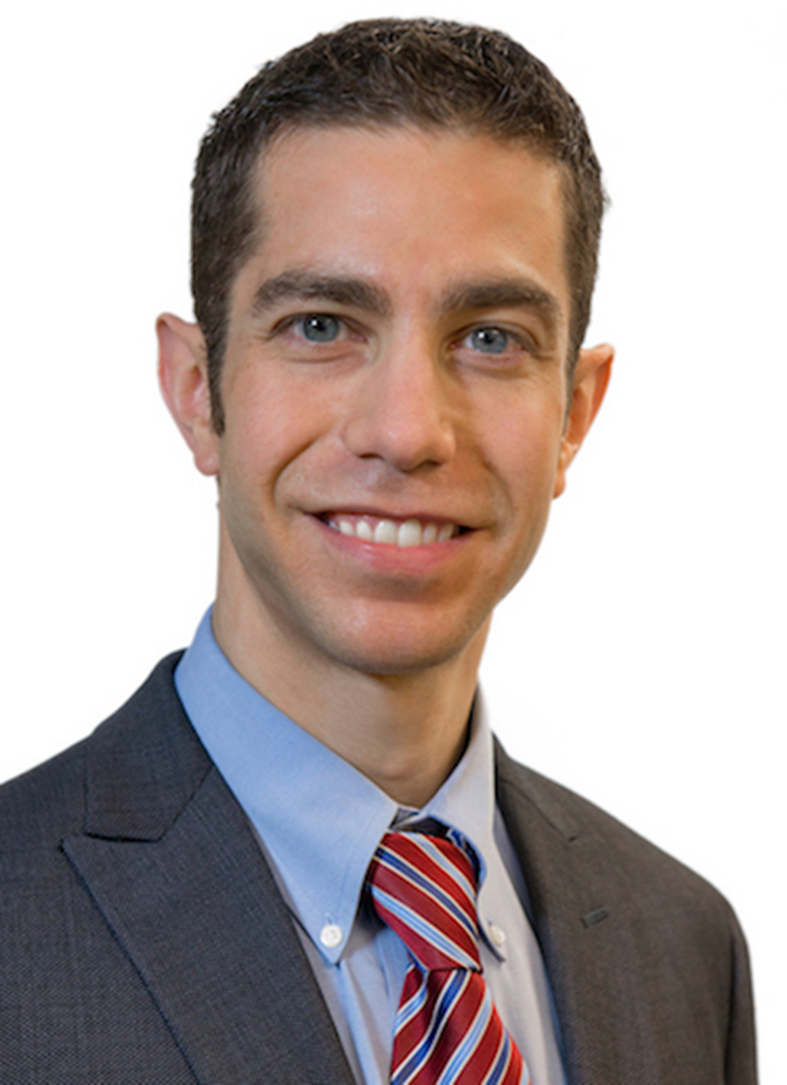

The COVID-19 pandemic presented innumerable challenges in medicine and notably left an indelible impact on trainees and training programs. Program directors (PDs) were tasked with leading during times of uncertainty, adapting to rapidly evolving guidelines and protocols, and also prioritizing trainees’ physical and mental health and safety. Previous surveys conducted by the American College of Cardiology (ACC) PD Survey Group focused on perceptions and utilization of the Core Cardiovascular Training Statement, diversity in cardiovascular (CV) training programs, and PD burnout and well-being.^1^, ^2^, ^3^ However, the timing of this survey captured a unique snapshot in medical education history, revealing the consequences and silver linings of a completely transformed and sometimes tumultuous educational era. Prior surveys during the COVID-19 era evaluated the impact on procedural volume and competencies,^4^^,^^5^ but this study is the first to assess the impact on general CV fellowship programs, providing insights to inform future training strategies and priorities.

The study by Cullen et al^6^ in this issue of *JACC: Advances* evaluated the educational and programmatic impact of the pandemic on general CV fellowship programs through the subjective lens of PDs. The authors describe the findings of the ACC PD Survey administered between April and July 2021, during the early phases of the COVID-19 pandemic when massive spikes in acute COVID-19 illness continued to occur. PDs from 139 U.S. programs completed the survey, with the majority (58%) from university-based centers. The study revealed that most PDs described a negative impact on clinical education in areas such as echocardiography, nuclear imaging, and outpatient general cardiology, while inpatient education was perceived to be less negatively affected. Virtual education platforms were rapidly adopted, and while most PDs noted improved online attendance, engagement and interaction were often reduced. Well-being and burnout among fellows were adversely impacted, according to the vast majority of PDs, with negative effects on research productivity and mentorship relationships. Finally, as recruitment processes transitioned to virtual structures, programs attracted more competitive applicants, along with notably higher applicant volumes.

There are several strengths of this study that merit highlighting. First, the exceptional survey response rate of 54% is noteworthy, especially in a climate saturated with surveys, and comparable to the authors' previous surveys.^1^, ^2^, ^3^ The results are also quite generalizable across program type, size, and region. Additionally, the questions posed were thoughtful, clear, and not overly burdensome, facilitating the easy completion of the entire survey and providing a wealth of useful information.

While many responses were unsurprising, the publication of this survey offers tangible data validating what many PDs experienced during the initial days of the pandemic. As hospitals faced unprecedented stress levels, residencies and fellowships nationwide had to adapt their core structures. This study reveals the decline in procedure numbers, research productivity, and the surge in burnout among trainees and others. The knowledge that most programs encountered similar challenges during the pandemic's peak offers a sense of solidarity among PDs across the country.

Perhaps most notably, this study is the only comprehensive survey to capture the perspective of CV PDs during the COVID-19 pandemic, and the data significantly contributes to the literature. As outlined in the manuscript, PDs have unique oversight of fellowship training, and their experiences during this crisis are invaluable for understanding what strategies were effective and what lessons can guide future responses to similar events.

While the pandemic undoubtedly inflicted significant hardship on trainees, the authors' emphasis on negative consequences overlooks several noteworthy findings warranting further discussion. For instance, while nearly half of respondents felt that virtual conferences negatively impacted fellow education, 22% believed the virtual format actually enhanced education. Higher attendance among fellows and faculty may partially explain this response, but there are likely other contributing factors. Moreover, while overall well-being declined, 25% of respondents felt the pandemic improved access to resources for addressing burnout, possibly due to increased attention to this issue.

The authors also underscore the pandemic's adverse effects on research mentorship and job prospects after fellowship, but a sizable portion of respondents felt there was no significant impact. Similarly, although the authors note “mixed perceptions” regarding the impact of virtual interviews on gender and race, the majority of PDs believed it either improved or had a neutral impact on gender and racial diversity among applicants, a crucial finding influencing decisions about continuing the virtual format.

A key limitation of this study is the applicability and reproducibility of its results. As with any survey, responses reflect the period during which the survey was conducted. Given the survey's early distribution in the pandemic, perceptions of how COVID-19 affected fellowship training likely evolved rapidly over a short period. For instance, would 92% of respondents still view virtual interviews as temporary if surveyed a year later? Therefore, the timing and stage of the pandemic when the survey was conducted must be considered when assessing how these findings may shape the future of fellowship education.

As the authors acknowledge, the extent to which the pandemic influenced medical knowledge and overall competency remains to be fully understood. The suggestion that initial certification examination scores in CV disease decreased as a result of the COVID-19 impact on training is debatable, as the downward trend began before the pandemic.^7^ What is clear is that many of the virtual tools that initially emerged out of necessity during the pandemic have now become integrated in fellowship programs. Virtual interviews were a sudden shift in the fall of 2020 and have remained standard for the past 4 recruitment seasons. Drawbacks of virtual interviews remain similar to those noted in the survey (Figure 1). Nevertheless, the advantages are obvious. Applicants can save significant time and expense associated with traveling to multiple programs. Moreover, reducing the numerous airline flights associated with interviewing is a vital step in lowering our carbon footprint and mitigating climate change impacts. Additionally, as previously mentioned, the survey noted increases in racial and gender diversity, a finding necessitating additional investigation for validation.

**Figure 1:**
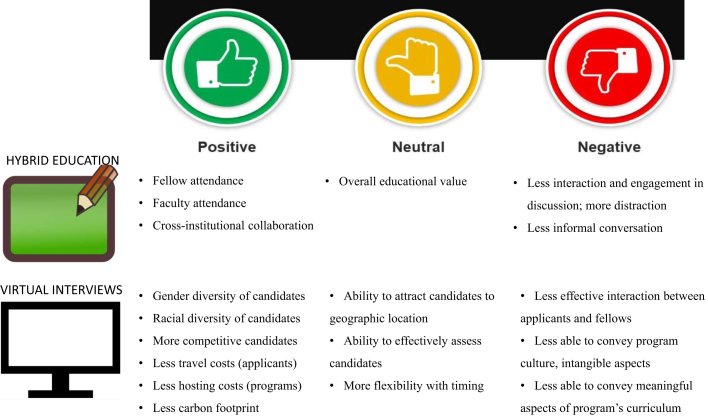
Hybrid Educational Experiences and Virtual Interviews Are 2 Major Changes That Have Remained Integral Postpandemic

Virtual education methods have also become the mainstay, as predicted by the majority of PDs even in the early days of the pandemic (Figure 1). Educational didactics and conferences for fellows that are solely online risk being less interactive and therefore less educational. However, the advantages of live-streaming conferences to satellite locations, recording lectures for later viewing, and aiding trainees in achieving a work-life balance by offering greater flexibility have led many programs to continue to offer a virtual learning option. The expansion of this format also allows programs to supplement subspecialized niche educational opportunities (ie, sports cardiology, cardio-obstetrics, critical care cardiology, and so on) through webinars and online conferences offered outside their institutions.

The ACC PD surveys offer valuable insight into the landscape of fellowship educational training within our specialty. The rapid and significant changes to medicine and education during the COVID-19 pandemic warrant thorough study and reflection. Fellowship administrators, PDs, and fellows, in particular, demonstrated remarkable resilience and adaptability, and training programs maintained high-quality education even in the midst of this unprecedented crisis. The authors of this study are commended for thoughtfully examining the acute educational impact of the pandemic. Although we hope to never face another global pandemic, we recognize that future challenges will inevitably arise, and we are confident our CV fellowship training programs will continue to pivot, adapt, reflect, and learn.

## Funding support and author disclosures

The authors have reported that they have no relationships relevant to the contents of this paper to disclose.
